# Nailing the Diagnosis: Onychotillomania in Patients With Artificial Nails—An Underrecognized Phenomenon?

**DOI:** 10.7759/cureus.24737

**Published:** 2022-05-04

**Authors:** Philip R Cohen, Razelle Kurzrock

**Affiliations:** 1 Dermatology, University of California, Davis Medical Center, Sacramento, USA; 2 Medicine, Medical College of Wisconsin Cancer Center and Genome Sciences and Precision Medicine Center, Milwaukee, USA

**Keywords:** repetitive, pick, onychotillomania, nail, focused, disorder, body, behavior, artificial, acrylic

## Abstract

Artificial nails are an essential component of nail cosmetics. The artificial nails are either preformed and glued onto the existing nail plate or they are custom made by applying a polymerizing mixture to the existing nail plate and overlying the template with a paintbrush that is subsequently allowed to harden into an acrylic nail. Artificial nails require regular maintenance. Onychotillomania is a body-focused repetitive disorder in which the person is usually aware that they are picking at their nail and/or the surrounding soft tissue. A woman with onychotillomania affecting her artificial nails is described; although this may be a relatively common occurrence, additional reports of artificial nail-associated onychotillomania were not able to be retrieved from the medical literature. The woman was not only aware that she picked at her artificial nails, but also realized that the action might result in adverse events to her natural nails and the corresponding digits. She desired no interventions for her nail-associated repetitive behavior and continued to regularly visit the nail salon for the application of new artificial custom acrylic nails. The acronym ANASON is introduced to define the condition of artificial nail-associated onychotillomania.

## Introduction

Nail cosmetics are a means of self-expression utilized by many individuals. In addition to polish and jewelry that can be applied or attached to the nail plate, artificial nails can be used as a nail adornment. Potential adverse effects associated with artificial nails include allergic contact dermatitis and infections [1-4].

Onychotillomania is a body-focused repetitive disorder associated with nail picking. It can occur as an isolated condition; alternatively, it can be associated with other nail conditions such as habit-tic nail deformity or onychophagia, or both. In addition, onychotillomania can concurrently be present in patients with body-focused repetitive disorders affecting the skin (such as dermatodaxia) or hair (such as trichotillomania) [5-14].

Onychotillomania, to the best of our knowledge, has not been reported in individuals with artificial nails. However, we suspect this phenomenon is more common than the paucity of case reports would imply. A 67-year-old woman who habitually picks off her artificial nails is described and the features of her artificial nail-associated onychotillomania are summarized.

## Case presentation

A 67-year-old woman presented with multiple dystrophic fingernails on both of her hands. She would visit a nail salon every week or every other week and artificial custom acrylic nails would be applied to all her fingernails. During the subsequent week, she would randomly pick at the artificial nails until they had been pulled off.

The patient began to have artificial nails applied to her fingers 15 years earlier. Prior to initiating the application of the acrylic nails, she never picked at her fingernails. The habit of nail picking originated after the artificial fingernails had been applied; as the acrylic nail would begin to detach from the underlying natural nail plate, she had the urge to pick at the artificial nail until the acrylic nail had completely detached. During the past 15 years, her nail picking habit has intensified.

**Figure 1:**
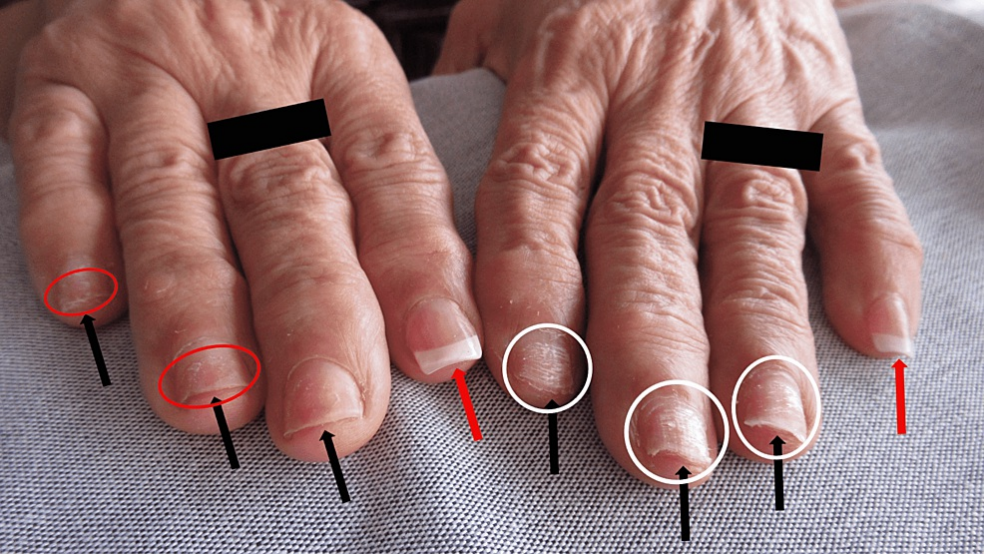
Onychotillomania of artificial nails The fingers of a 67-year-old woman demonstrate artificial nail-associated onychotillomania (ANASON). The patient has picked off the artificial nails on several digits (black arrows) of her right (third, fourth, and fifth fingers) and left (second, third, and fourth fingers) hands; the surface of the affected nail plates is dystrophic and the distal edges are irregular. The patient’s natural nail plates are rough (trachyonychia) with horizontal shedding (onychoschizia) (red ovals). In addition, there are spotty and transverse white patches (leukonychia) (white ovals). The artificial nails remain intact (red arrows) on the right index finger and the left little finger. The black rectangles obscure rings that the patient was wearing on her right third and left fourth fingers.

**Figure 2:**
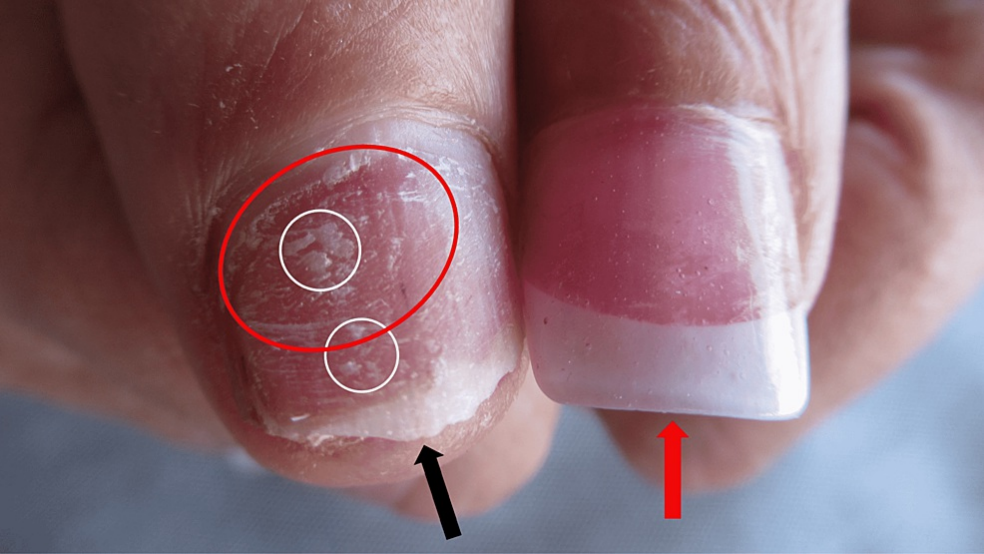
Artificial nail-associated onychotillomania (ANASON) The artificial nail previously present on the right thumb of the patient has been picked off (black arrow). The surface of the patient’s natural nail plate has spotty leukonychia (white patches) (white ovals), onychoschizia (horizontal shedding) (red oval), and trachyonychia (roughness); in addition, the free edge that is not smooth. In contrast, the artificial nail is attached to the left thumb (red arrow).

Cutaneous examination of her hands showed multiple digits with and without artificial nails. The artificial nails on her right third, fourth, and fifth fingers were missing; in addition, those on her left second, third, and fourth fingers were also absent. The surface of the affected nail plates was dystrophic and the distal edges are irregular (Figure 1). Similarly, the right thumb did not have an artificial nail; its surface was rough (trachyonychia) with spotty white areas (leukonychia) and a non-smooth free edge (Figure 2). Intact artificial nails were noted on the right index finger, the left little finger, and the left thumb (Figures 1-2).

**Figure 3:**
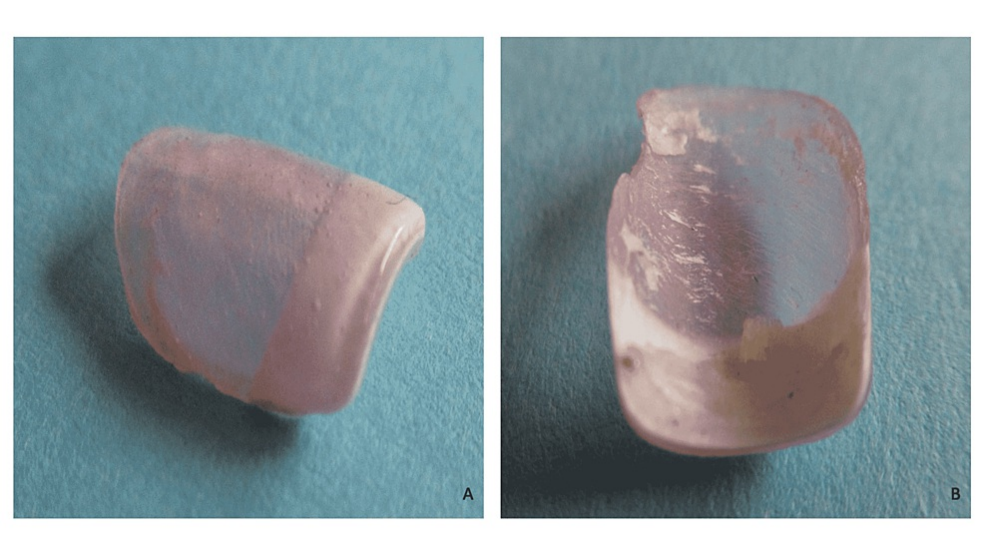
An artificial nail The superior (A) and underside (B) views of an artificial nail that the patient had picked off show polish on the superior surface (A) and residual adhesive with small fragments of nail plate on the underside surface (B).

The patient provided one of the artificial acrylic nails that she had picked off. The superior surface showed nail polish. The underside surface demonstrated residual adhesive with small fragments of the nail plate (Figure 3).

Correlation of the history and clinical presentation established the diagnosis of onychotillomania of the artificial nails. The patient was aware of her aggressive artificial nail-associated body-focused repetitive behavior and its harmful effect on the natural underlying nail; however, she experienced soothing comfort during her nail picking and their traumatic removal. She declined any discussion regarding an attempt to modify her behavior and elected to continue to visit the nail salon either every week or every other week for the application of new artificial nails on her fingers.

## Discussion

Nail adornment is a common practice predominantly by women; however, it also can be observed in men. Artificial nails are a form of nail cosmetics. There are two types of artificial nails: preformed artificial nails and custom artificial nails [1,2].

Preformed artificial nails, also referred to as press-on nails, are designed for short-term use, usually less than one week. They have a methacrylate-based adhesive on the inner surface. The shape and length of the artificial nail can be modified by trimming or cutting after the nail has been pressed onto the natural nail. Horizontal nail shedding (onychoschizia) and nail pitting result from their traumatic removal. In addition, artificial nails may create adverse effects such as contact dermatitis not only in nail salon workers but also in clients who are allergic to methacrylate [1-3].

Custom artificial nails, also known as nail sculptures, use a custom-designed mixture for nail elongation. Similar to the artificial nails applied to the woman in this report, the artificial nail is sculpted to fit the client’s fingertip. The acrylic nail plate is created by mixing powdered methacrylate polymer with either liquid ethyl methacrylate or isobutyl methacrylate and benzoyl peroxide (as an accelerant); the mixture polymerizes and hardens into an acrylic in 7-9 minutes. Polymerization can be slowed by adding either hydroquinone, monoethyl ether of hydroquinone, or pyrogallol. After a template has been placed on the person’s nail plate, a paintbrush is used to apply the mixed, yet non-hardened, acrylic to the nail plate and overlying template. Custom artificial nails need professional maintenance approximately every three weeks; otherwise, nail shedding (onychoschizia) and damage to the natural nail plate may occur [1-3].

Artificial nails have also been associated with bacterial and fungal infections. These can affect the patient who is wearing the nails. In addition, hospitalized patients are at increased risk of acquiring infection from individuals wearing artificial nails [4].

Onychotillomania is a condition in which the patient is picking their nails. The term originates from the Greek onycho (meaning nail), tillo (meaning to pull), and mania (meaning frenzy or madness). Earlier reports of idiopathic onychotillomania observed the condition to occur in patients with psychiatric disorders. However, psychiatric conditions have not been implicated in patients with artificial nail-associated onychotillomania (ANASON) [6,7,10,11].

Onychotillomania is not a frequently observed disorder. The Wisconsin Psychocutaneous Clinic gathered data from their one-day-a-week clinic from May 2002 to February 2018; there were 808 referrals: 619 women and 189 men. Skin picking disorder was the most common diagnosis (in 417 patients); however, at least 11 of the 15 individuals with other specified obsessive-compulsive disorders had onychotillomania. Several of the patients had more than one diagnosis: 10 patients had both dermatodaxia and onychotillomania and one patient picked at their skin, nails, and hair [11,15,16].

Onychotillomania often begins in childhood or adolescents; however, one group of investigators showed that the patient’s average age at diagnosis was 47.5 years. Onychotillomania is more common in men. Also, most individuals with onychotillomania are aware of their nail-focused repetitive behavior [10,11].

Dermoscopy is a noninvasive imaging method that can be used in the evaluation of nail-associated body-focused repetitive behaviors such as onychotillomania and onychophagia. Two investigators demonstrated that characteristic dermoscopic findings of onychotillomania-which were not observed in other nail diseases-included the absence of the nail plate with multiple obliquely oriented nail bed hemorrhages nail bed gray pigmentation and the presence of wavy lines [17]. Subsequently, another group of researchers that evaluated the clinical and dermoscopic features of onychotillomania and onychophagia, observed that Beau lines (horizontal deep grooves that extend across the nail plate and are caused by a limited injury to the nail matrix resulting in a temporary arrest of nail keratin synthesis) and washboard-like nail (a series of ridges that extend across the nail plate giving it the appearance of a washboard) were distinctive features for onychotillomania [18].

Recently, onychotillomania was described in a 32-year-old homeless man with comorbid major depressive disorder and methamphetamine use disorder. Two months after becoming homeless, he developed the nail picking behavior that involved both his fingernails and toenails; he presented with short painful nails, nail fold erosions, and paronychia. In addition to using his fingernails, he also used a nail file, tweezers, and a nail cutter to pick, pull, and cut his nails [19].

The reported woman with artificial nail-associated onychotillomania presented with multiple absent acrylic nail plates. In addition, secondary to the traumatic removal of the artificial nails, her natural nail plates had pitting, spotty leukonychia, and horizontal nail shedding. In contrast, patients with idiopathic onychotillomania present with nail plates that range from being dystrophic to absent; other clinical abnormalities of the affected nail and surrounding soft tissue of the nail unit in patients with onychotillomania are listed in Table 1 [10,13,18].

**Table 1:** Clinical presentations of onychotillomania ^a^These include herpetic whitlow (caused by herpes simplex virus), molluscum contagiosum (caused by pox virus), and verruca vulgaris (caused by human papillomavirus).

Morphology
Bacterial infection of the nail fold
Candida infection of the nail fold
Central furrow with transverse parallel ridges of the nail plate
Cuticle loss
Digit susceptible to viral infections^a^
Macrolunula
Melanonychia
Nail bed exposure
Nail fold hyperpigmentation
Nail fold and hyponychium trauma
Acute paronychia
Chronic paronychia
Erythema
Erosions
Excoriations
Nail plate absence
Nail plate atrophy
Nail plate pitting
Nail plate shortening
Onychoschizia (nail plate horizontal shedding)
Pterygium formation
Subungual hematoma
Trachyonychia (rough nail plate)

The management of onychotillomania incorporates techniques that do not use drugs or treatments that include medications, or both. Cognitive behavioral therapy, habit-removal therapy, stimulus control procedures, and occlusive barriers are non-pharmacologic therapeutic interventions [11-13,20]. For example, onychotillomania treatment for the 32-year-old homeless man was multifactorial; he not only received counseling and distracting tools so that he would avoid nail picking but also was provided with resources to treat his substance use disorder [19].

The reported woman with artificial nail-associated onychotillomania was cognizant of her nail picking. Indeed, even after additional discussion regarding the potential adverse events associated with her behavior, she still experienced comfort by removing the less-adherent artificial nails. In addition, she returned to the nail salon either weekly or every second week to acquire new artificial nails.

We postulate that the presentation of onychotillomania associated with artificial nails is not uncommon. The reported patient’s nail picking behavior originated only after she started having acrylic nails applied to her natural fingernails and the habit has subsequently intensified. Yet, we are unaware of any previous reports of this phenomenon. Therefore, we have introduced an acronym for artificial nail-associated onychotillomania: ANASON (Table 2).

**Table 2:** Derivation of ANASON acronym ANASON: Artificial nail-associated onychotillomania ^a^ANASON is the Turkish translation for the English word anise. Anise—also referred to as aniseed, *Pimpinella anisom*, or rarely as anix—is a herbaceous annual flowering plant of the parsley (Apiaceae) family and has a black licorice flavor.

Letter^a^	Source
A	The first letter of the word artificial.
N	The first letter of the word nail-associated.
A	The fifth letter of the word nail-associated.
S	The sixth letter of the word nail-associated.
O	The first letter of the word onychotillomania.
N	The second letter of the word onychotillomania.

## Conclusions

Artificial nails-either preformed or custom-are a component of nail cosmetics that are used by many people. Onychotillomania, also referred to as nail picking, is a body-focused repetitive disorder; it can occur on its own or it can be associated with other nail disorders such as habit-tic nail deformity and/or onychophagia or other body-focused repetitive disorders such as dermatodaxia and trichotillomania. A woman with onychotillomania involving her artificial nails is described; in spite of her awareness and insight into the potential adverse events associated with her repetitive behavior, she continued to experience comfort picking off her custom nails and would frequently return to the nail salon for the application of new artificial acrylic nails. We introduce the acronym ANASON to define those individuals with artificial nail-associated onychotillomania.

## References

[1] Draelos ZD (2021). Nail cosmetics and adornment. Dermatol Clin.

[2] Madnani NA, Khan KJ (2012). Nail cosmetics. Indian J Dermatol Venereol Leprol.

[3] Voller LM, Warshaw EM (2020). Acrylates: new sources and new allergens. Clin Exp Dermatol.

[4] Toles A (2002). Artificial nails: are they putting patients at risk? A review of the research. J Pediatr Oncol Nurs.

[5] Colver GB (1987). Onychotillomania. Br J Dermatol.

[6] Inglese M, Haley HR, Elewski BE (2004). Onychotillomania: 2 case reports. Cutis.

[7] Reese JM, Hudacek KD, Rubin AI (2013). Onychotillomania: clinicopathologic correlations. J Cutan Pathol.

[8] Pacan P, Grzesiak M, Reich A, Kantorska-Janiec M, Szepietowski JC (2014). Onychophagia and onychotillomania: prevalence, clinical picture and comorbidities. Acta Derm Venereol.

[9] Snorrason I, Woods DW (2014). Nail picking disorder (onychotillomania): a case report. J Anxiety Disord.

[10] Rieder EA, Tosti A (2016). Onychotillomania: an underrecognized disorder. J Am Acad Dermatol.

[11] Singal A, Daulatabad D (2017). Nail tic disorders: manifestations, pathogenesis and management. Indian J Dermatol Venereol Leprol.

[12] Magid M, Mennella C, Kuhn H, Stamu-O'Brien C, Kroumpouzos G (2017). Onychophagia and onychotillomania can be effectively managed. J Am Acad Dermatol.

[13] Halteh P, Scher RK, Lipner SR (2017). Onychotillomania: diagnosis and management. Am J Clin Dermatol.

[14] Sidiropoulou P, Sgouros D, Theodoropoulos K, Katoulis A, Rigopoulos D (2019). Onychotillomania: a chameleon-like disorder: case report and review of literature. Skin Appendage Disord.

[15] Mostaghimi L (2021). Psychocutaneous Medicine Clinic: Wisconsin experience. J Acad Consult Liaison Psychiatry.

[16] Cohen PR (2022). Skin biter: dermatodaxia revisited. Cureus.

[17] Maddy AJ, Tosti A (2018). Dermoscopic features of onychotillomania: a study of 36 cases. J Am Acad Dermatol.

[18] Chen Y, Pradhan S, Lyu L, Xue S (2022). Clinical and dermoscopic characteristics of onychophagia and onychotillomania: a retrospective study of 63 cases. Clin Exp Dermatol.

[19] Rasul TF, Gulraiz S, Henderson A (2022). Onychotillomania in the setting of homelessness. Cureus.

[20] Cohen PR (2022). Nail-associated body-focused repetitive behaviors: habit-tic nail deformity, onychophagia, and onychotillomania. Cureus.

